# Recruitment and characteristics of participants in trials of physical activity for adults aged 45 years and above in general practice: a systematic review

**DOI:** 10.1093/fampra/cmac128

**Published:** 2022-12-06

**Authors:** Richard McNamara, Kimberly Davy, Vikram Niranjan, Andrew O’Regan

**Affiliations:** University of Limerick, Health Research Institute, School of Medicine, Limerick, Ireland; University of Limerick, Health Research Institute, School of Medicine, Limerick, Ireland; School of Public Health, Physiotherapy and Sports Science, University College Dublin, Dublin, Ireland; University of Limerick, Health Research Institute, School of Medicine, Limerick, Ireland

**Keywords:** exercise, general practice, physical activity, randomized control trials, recruitment, systematic review

## Abstract

**Background:**

General practice is well situated to promote physical activity (PA), but with PA levels declining after 45 years of age, often those who are most likely to benefit from interventions tend to be the least likely recruited to participate in research.

**Aims and rationale:**

The aim of this study was to investigate recruitment and reporting of participant demographics in PA trials for adults aged 45 years and above. Specific objectives were: (i) to examine the reporting of demographics of participants; (ii) to investigate the strategies used to recruit these participants; and, (iii) to examine the efficiency of recruitment strategies.

**Methods:**

Seven databases were searched, including: PubMed, CINAHL, the Cochrane Library Register of Controlled Trials, Embase, Scopus, PsycINFO, and Web of Science. Only randomized control trials involving adults 45 years old or older recruited through primary care were included. The PRISMA framework for systematic review was followed, which involved 2 researchers independently conducting title, abstract, and full article screening. Tools for data extraction and synthesis were adapted from previous work on inclusivity in recruitment.

**Results:**

The searches retrieved 3,491 studies of which 12 were included for review. Sample size of the studies ranged from 31 to 1,366, with a total of 6,042 participants of which 57% were female. Of 101 participating practices, 1 was reported as rural. Reporting of recruitment lacked detail—only 6 studies outlined how practices were recruited. 11/12 studies involved a database or chart review to identify participants that met the inclusion criteria, followed by a letter of invitation sent to those people. The studies with higher recruitment efficiency ratios each employed more than 1 recruitment strategy, e.g. opportunistic invitations and telephone calls.

**Conclusion:**

This systematic review has presented deficits in the reporting of both demographics and recruitment. Future research should aim for a standardized approach to reporting.

**Key messages**

- Reporting of participant demographics is mixed.
- There is no consistency for reporting socioeconomic factors.
- Even studies with high reporting scores had low population diversity.
- Reporting of recruitment was sparse especially methods for practice enrolment.
- Efficiency of recruitment was higher when multiple strategies were used.
- Future research should aim to standardize recruitment and demographic reporting.

## Introduction

Being physically active is protective against chronic illnesses, including cancer, heart disease, dementia, and depression, as well as all-cause and cardiovascular mortality. However, the WHO has reported that, in high-income countries, over one quarter of adults are not active enough, and, in some countries, levels of physical inactivity (PiA) are as high as 70%,^1^ with estimated costs of $67.5 (USD) billion worldwide.^2^ Consequently, the WHO declared PiA a pandemic, it being the fourth leading cause of death worldwide.^3^ A decade of behavioural change endeavour has not addressed this, and the COVID-19 pandemic has exacerbated an already alarming public health concern.^4^

The WHO Guidelines for Physical Activity and Sedentary Behaviour recommend that adults should achieve at least 150 min of moderate-intensity or 75 min of vigorous-intensity physical activity (PA) each week, while stressing that any activity is better than none, even if minimum PA targets are not reached.^5^ Studies have reported an inverse dose–response relationship between PA and mortality and that small doses of PA could alleviate mortality risk, and boost primary and secondary prevention of chronic illness^6,7^; this relationship is curvilinear, with the greatest health benefits stemming from relatively small levels of PA.^8^ Chronic illnesses, particularly, cardiovascular disease, cancer, chronic lung disease, and diabetes, accounted for 71% of deaths (57 million) in 2016,^9^ and are the leading cause of mortality worldwide; 10 million of these deaths could be avoided through evidence-based approaches, including PA promotion through community-based programmes aimed at behavioural change.^10^

Chronic illness prevalence is high in general practice populations,^10,11^ and research reports that the general practice may be an effective environment for PA promotion among adults^12^; through repeated contacts and continuity of relationships, general practitioners can identify patients who are insufficiently active.^13^ Socioeconomic and health status are factors that affect PA levels.^14,15^ Recruitment to PA research has been challenging: for example, men and smokers are harder to reach participants to PA interventions,^16^ with selection bias towards those that are better off and better educated.^17^ The phenomenon of the availability of good medical care tending to vary inversely with the need for it, described by GP Dr Julian Tudor-Hart in the “inverse care law,”^18^ remains relevant.^19^ The inverse care law may also apply to PA promotion,^20^ and the WHO guideline development group recommend further research into how health and socioeconomic factors moderate PA and health outcomes.^21^

Research reports that PA levels decline after 45 years of age, and a knowledge gap exists in the literature as to how adults aged 45 years and over are recruited to PA trials from general practice.^22^ Similarly, no study of PA trials in the general practice setting has reviewed the profiles of participants across the trials. It is important for clinicians to know the demographics of study populations to decide if the findings are relevant to their patient population. Therefore, the aim of this systematic review was to systematically review randomized controlled trials (RCTs) of PA interventions for adults aged 45 years and older conducted in general practice to investigate participant demographics and recruitment.

## Objectives

1. To examine the reporting of demographics of participants aged 45 years and older in PA trials in general practice;
2. To investigate the strategies used to recruit these participants; and
3. To examine the efficiency of such recruitment strategies.

## Methods

In accordance with best practice for systematic reviews, the full protocol for this systematic review was registered with the PROSPERO international prospective register of systematic reviews database (registration number CRD42020194338, 2020/07/27).^23^ Findings are reported according to PRISMA (Preferred Reporting Items for Systematic reviews and Meta-Analyses) guidelines.^24^

### Search strategy

The strategy and search protocol were devised with the help of a librarian at the University of Limerick (Supplementary Data 1). Searches were conducted by RM and KD, under the supervision of AOR, in July 2020 and updated in November 2021; quality control was ensured by a fourth researcher (VN). No limitation was put on the year of publication. Seven electronic literature databases were searched: PubMed, CINAHL, the Cochrane Library Register of Controlled Trials, Embase, Scopus, PsycINFO, and Web of Science. Reference lists of included studies were also screened for relevant papers. The search terms were: (family practice OR general practice OR primary care) AND (physical activity OR exercise) AND (adult OR older adult) AND (recruit*).

### Study selection

#### Inclusion and exclusion criteria

RCTs were included, as per the Patient, Intervention, Comparison and Outcome (PICO) strategy, involving adults 45 years old or older recruited through primary care. The intervention of interest was PA compared with none or alternative PA interventions, where the primary outcome was increased PA levels or improved health. The study was restricted to middle-aged adults and older persons aged over 45 years. This age cut-off was chosen as it has been identified as an age when PA begins to decline.^22^ Three of the 7 electronic databases (PubMed, Embase, and CINAHL) interrogated use age filters that define middle age as beginning at 45 years. Studies that did not have PA as the sole intervention and did not have improved PA levels as the primary outcome were excluded, as were pilot and feasibility studies. Studies in languages other than English or that were published in locations other than peer-reviewed journals were excluded.

#### Screening process

The reference citation manager Endnote was used to assist the screening process. The PRISMA approach involves systematic screening of titles, abstracts, and full text, which were conducted independently by RM, KD, and AOR. Consensus on screening was reached through a process of discussion; where uncertainty existed, the full paper was read. Uncertainty regarding study eligibility was resolved by VN.

### Data extraction

Data extraction was conducted independently by 2 authors (RM and KD) and AOR checked for consensus. A standardized form was used for initial data extraction, facilitating the recording of study title, authors, year, location, population, outcome, comparator, duration, and follow-up. The research question of this study relates to recruitment and study population, so brief synopses only were included on data not relating to these aspects of the trials under review. However, as detail regarding trial participants and recruitment is often contained in study protocols, other papers relating to the study and online reports, where relevant such papers were read and information in them was included in this review.

### Study population

A data extraction tool was designed for study population, adapted from metrics used by Foster et al.,^25^ O’Neill et al.,^26^ and Attwood et al.^27^ A 10-point scale was devised based on whether the following population descriptors were reported at baseline: gender (1 point); inactive (defined as not reaching PA targets; 1 point); place of residence (urban vs. rural, deprivation index; 1 point each); ethnicity or minority groups reported (1 point); socioeconomic group (income, education; 1 point each); domestic status (marital status or whether living alone); disability (chronic condition, mental illness, or multimorbidity; 1 point); and smoking status (1 point). The reporting quality for study population is presented in Table 1.

**Table 1.** Description of participant demographics.

Author, year, location	Gender % female (1 point)	Inactive population (1 point)	Participant residence or practice location Urban/rural (1 point) Deprivation index (1 point)	Ethnicity or minority groups (1 point)	Socioeconomic group reported: Income (1 point) Education (1 point)	Social status: Marital status or whether living alone (1 point)	Disability (chronic condition, mental illness, multimorbidity) (1 point) Smoking status (1 point)	Demographic reporting score
Stevens et al., 1998, United Kingdom^28^	63% (449)	97% were classified as sedentary or low active.	Two urban practices	Ethnic minorities reported, *n* = 13% intervention, *n* = 17% control	55% were working (no income or type of work described). No data on retirement status. 36% had not completed formal secondary school education.	N/R	N/R 18% were smokers	7
Halbert et al., 2000, Australia^29^	69% (207)	N/R	Two urban practices	No data on ethnic minorities but 72% were born in Australia	36% were currently employed but no data on retirement status.	77% were married. No data on whether living alone.	38% had a chronic condition but no data on multimorbidity or mental illness. Mean visits 4.4 6% were smokers	6
Petrella et al., 2003, Canada^30^	49% (117)	No data on baseline PA levels.	Three urban, 1 rural practice	No data on ethnicity	19% were on very low incomes (<$10,000); 42% had <12 years of formal education.	56% were single or widowed. No data on whether living alone.	55% had multimorbidity but no data on mental illness.	6
Tully et al., 2005, Northern Ireland^31^	N/R	Only physically inactive people were included.	Three urban practices	No data on ethnicity	N/R	N/R	Only subjects with no significant chronic condition were included.	3
Kolt et al., 2007, New Zealand^32^	66% (123)	24% of participants were already achieving 150 min of MVPA per week.	Three urban practices	97% were classified as New Zealand European	85% were retired. There was no data on income. 56% had left education before or at completion of secondary school. 75% owned and drove a car.	49% were married or living with a partner.	No data on chronic conditions. Self-report questionnaires relating to physical and mental health.	7
Kolt et al., 2012, New Zealand^33^	54% (178)	Inclusion criteria included low-active older adults.	Ten urban practices	97% were classified as New Zealand European	78% were retired. No data on income. 45% had left education before or at completion of secondary school. 92% owned and drove a car.	63% were married or living with a partner.	43% were taking cardiovascular medications.	8
Devi et al., 2014, United Kingdom^34^	26% (24)	Participants tended to be low-active.	Nine practices	86 (93%) were classified as White British	2 (2%) were unemployed. 50 (53%) were retired. No income/education data.	No data on marital status.	Inclusion criterion was diagnosis of angina. People on antidepressant/anxiolytic medication were excluded. No data on comorbidity. Some disease perception data. 9% were smokers	6
Harris et al., 2015, United Kingdom^35^	54% (160)	No distinction was made based on PA levels at baseline.	Three practices 29 (10%) came from the most socially deprived areas. 218 (73%) came from the least socially deprived areas. Used national indices of social deprivation.	97% White	59% (175) were retired. 42% (126) had tertiary education.	81% (240) were married.	91 (30%) had no chronic illness. 178 (60%) had 1–2. 29 (10%) had 3 or more chronic illnesses. 5% were smokers	8
Iliffe et al., 2015, United Kingdom^36^	62% (782)	No distinction was made based on PA levels at baseline.	Forty-three practices from 3 cities, with practice-level deprivation indices.	86% were White and 34 different languages were reported as first language	N/R	N/R	On average, participants had 1.7 chronic conditions and 3.7 regular medications.	5
Harris et al., 2018, United Kingdom^37^	64% (656)	Inactive participants only were recruited.	Six urban practices. 223 (22%) were from deprived areas. Indices of social deprivation were used.	790 (77%) were White	299 (29%) were retired. 573 (56%) were in full- or part-time work. 147 (14%) were in current/previous routine or manual occupations.	658 (64%) were married. No data on living alone.	542 (53%) had 1 or 2 chronic illnesses. 83 (8%) had 3 or more chronic conditions. 8% were smokers	9
Peacock et al., 2020, United Kingdom^38^	73 (36%)	Participants were all at medium or high risk of diabetes or cardiovascular disease.	Six practices. Indices of social deprivation were used.	180 (88%) were White	116 (57%) were retired. 63 (31%) left education at or before 16 years. 81 (40%) had a third-level qualification.	150 (74%) were married. No data on living alone.	40 (20%) were smokers. No data on comorbidities	8
Khunti et al., 2021, United Kingdom^39^	673 (49%)	Participants had prediabetes.	Two urban practices. Indices of social deprivation were used.	982 (72%) were White	145 (11%) were unemployed. 35% retired. 604 (44%) had a third-level qualification.	991 (73%) were married. No data on living alone.	35 (10%) were smokers. Data were collected on medications and illnesses related to diabetes.	10

### Recruitment

Data relating to quality of recruitment were extracted and synthesized using an adapted table based on previous research.^25,26^ Recruitment strategies, displayed in Table 2, reported the following: collaboration between research and clinical team, duration of recruitment, who conducted recruitment, mixture of recruitment strategies and recruitment of practices.

**Table 2.** Overview of recruitment strategies.

Stevens et al., 1998^28^	
How were practices recruited?	N/S
How were participants recruited?	An invitation letter containing a self-assessment questionnaire was sent to everyone on the surgery list within the specified age range
What support was available for the practices?	N/S
Duration of recruitment	N/S
Halbert (Kolt, Schofield et al., 2007), 2000^29^	
How were practices recruited?	GPs in each practice were invited by letter to participate. The invitation was followed by a visit from a member of the research team
How were participants recruited?	A researcher identified suitable participants from the practice databases in conjunction with a designated member of the practice team. Letters were sent to potential participants from the practice
What support was available for the practices?	The study coordinator helped with identification of suitable participants. Ongoing support was provided by the study coordinator
Duration of recruitment	N/S
Petrella et al., 2003^30^	
How were practices recruited?	Prior affiliation with a university
How were participants recruited?	Two methods: 1.Opportunistic word of mouth during clinics 2.A database of potential participants was generated, and they were phoned from the practice
What support was available for the practices?	N/S
Duration of recruitment	6 months
Tully (Halbert, Sllagy et al., 2000), 2005^31^	
How were practices recruited?	N/S
How were participants recruited?	Two methods: 1.GP invitation letter sent to potential participants to complete a postal questionnaire and to agree to being contacted by a research assistant 2.A subsample of 90 nonrespondents were telephoned by their GP after 3 weeks and given a second invitation
What support was available for the practices?	A research assistant helped the practice team with recruitment
Duration of recruitment	N/S
Kolt (Tully, Cupples et al., 2005), 2007^32^	
How were practices recruited?	N/S
How were participants recruited?	Suitable participants identified by chart review (practice team) and sent letter of invitation by GP and/or phone call from practice staff
What support was available for the practices?	A research assistant helped the practice team with recruitment
Duration of recruitment	18 months
Kolt (Kolt, Schofield et al., 2007), 2012^33^	
How were practices recruited?	GPs were faxed an invitation and those who responded were telephoned and visited by a member of the research team
How were participants recruited?	Potential participants were identified from the practice database, posted a letter signed by their GP, and those who were replied were phoned to give more information and perform eligibility screening
What support was available for the practices?	N/S
Duration of recruitment	17 months
Devi (Kolt, Schofield et al., 2012), 2014^34^	
How were practices recruited?	N/S
How were participants recruited?	Potential participants identified from coronary heart disease registers by GP or practice nurse. Recruitment was conducted sequentially by letter, telephone, and home visit
What support was available for the practices?	Research assistant aided recruitment
Duration of recruitment	N/S
Harris (Devi, Powell et al., 2014), 2015^35^	
How were practices recruited?	N/S
How were participants recruited?	GPs were involved in the identification of suitable participants from practice databases. Invitation letters were sent out to potential participants with follow-up letters after 6 weeks to nonresponders
What support was available for the practices?	Reference made to nurse training and support by study team
Duration of recruitment	11 months
Iliffe (Harris, Kerry et al., 2015), 2015^36^	
How were practices recruited?	Through a Primary Care Research Network
How were participants recruited?	Suitable participants identified by chart review (practice team) and sent letter of invitation by GP
What support was available for the practices?	A research assistant helped the practice team with recruitment
Duration of recruitment	27 months
Harris (Iliffe, Kendrick et al., 2015), 2018^37^	
How were practices recruited?	Practices were recruited through the Primary Care Research Network according to practice inclusion criteria
How were participants recruited?	Suitable participants identified by chart review (practice team) and sent letter of invitation by GP
What support was available for the practices?	Collaboration between the research and practice teams, and training for the practice team
Duration of recruitment	2 months
Peacock (Harris, Kerry et al., 2018), 2020^38^	
How were practices recruited?	N/S
How were participants recruited?	A database search was conducted to identify participants who met inclusion criteria. Potential participants were then contacted by a letter of identification from the GP
What support was available for the practices?	N/S
Duration of recruitment	N/S
Khunti (Peacock, Western et al., 2020), 2021^39^	
How were practices recruited?	Practices located in areas with large multiethnic areas were targeted
How were participants recruited?	1.The practice databases were searched followed by letter of invitation from GP to potential participants 2.Previous research databases were searched followed by a letter of invitation from the primary investigator responsible for the database
What support was available for the practices?	Training from the research team
Duration of recruitment	15 months

Abbreviation: N/S, not specified.

### Recruitment efficiency

Based on work by Foster et al.,^25^ extraction tables were created to document the following data: people available (the pool), people invited, people who attended screening, and the number who participated. Three ratios of efficiency were calculated: efficiency A, by dividing the number who started the trial into the number for the pool; efficiency B, by dividing the number who started into the number who were invited; and efficiency C, by dividing the number who started by the number who attended screening (Table 3).

**Table 3.** Recruitment efficiency.

	Pool	Invited	Attended screening	Participated	Efficiency A Started/pool	Efficiency B Started/invited	Efficiency C Started/attended screening
Stevens (Kolt, Schofield et al., 2007)^28^	N/S	2,253	827	714	N/A	32%	86%
Halbert (Stevens, Hillsdon et al., 1998)^29^	N/S	2,878	913	351	N/A	12%	38%
Petrella (Halbert, Sllagy et al., 2000)^30^	N/S	500	320	284	N/A	57%	89%
Tully (Petrella, Koval et al., 2003)^31^	527	50	50	31	6%	62%	62%
Kolt (Tully, Cupples et al., 2005)^32^	N/S	831	333	186	N/A	22.%	56%
Kolt (Kolt, Schofield et al., 2007)^33^	N/S	1,739	986	330	N/A	19%	33.5%
Devi et al., 2014^34^	N/S	612	131	95	N/A	15.5%	72.5%
Harris (Kolt, Schofield et al., 2012)^35^	3,679	988	N/S	298	8%	30%	N/A
Iliffe (Harris, Kerry et al., 2015)^36^	N/S	20,507	2,752	1,254	N/A	6%	46%
Harris (Iliffe, Kendrick et al., 2015)^37^	21,243	11,015	1,698	1,023	5%	9%	60%
Peacock (Harris, Kerry et al., 2018)^38^	N/S	1,484	533	204	N/A	13.7%	38%
Khunti (Peacock, Western et al., 2020)^39^	N/S	12,417	1,563	1,366	N/A	11%	87%

Abbreviations: N/A, not applicable; N/S, not specified.

## Results

### Study selection and overview

**Fig. 1.**
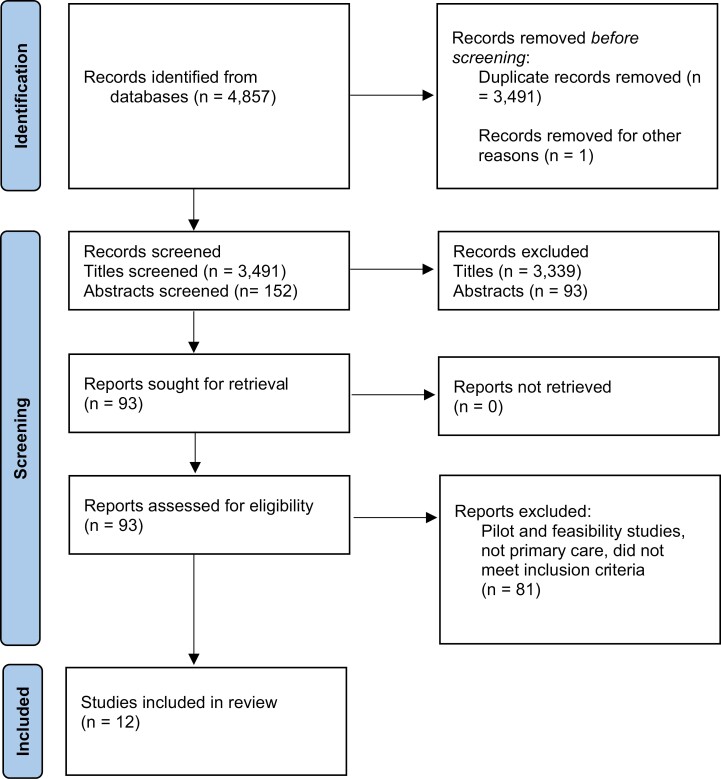
PRISMA flow diagram for study inclusion.

The searches identified 4,857 studies, and 12 of them were included in the review (Fig. 1), after title, abstract, and full text screening.^28–39^ The PRISMA screening process is outlined in Fig. 1; the most common reasons for excluding papers at full paper screening were age range, non-RCT, and settings other than general practice. The studies were published between 1998 and 2021. Six of the 12 included studies had associated published protocols, which were also read for the purposes of this review.^32–36,38,39^ One study had an associated publication on recruitment^29^; another was linked to a full online report that included details of recruitment and participant inclusion/exclusion criteria published online.^37^

In total, 101 general practices were recruited, yielding a total combined population of 6,042 participants (see Supplementary Material 1 for details on the studies and interventions). Eight studies were located in the United Kingdom,^28,31,34–39^ 3 in Australia and New Zealand,^29,32,33^ and 1 in Canada.^30^ Study participants were adults with a mean age range of 50–70 years. Three studies had over 1,000 participants,^36,37,39^ 1 had 714,^28^ 6 had between 100 and 400,^29,30,32,33,35,38^ and 2 had fewer than 100.^31,34^

### Reporting of participant demographics

Participant demographics are outlined in Table 1. Eleven studies reported gender and 7 of them had a majority of female participants.^28,29,32,33,35–37^ Of the studies that reported gender, a total of 3,442 (57%) were female. Five studies specified low-active participants as an inclusion criterion.^28,31,33,34,37^ A further 3 studies involved only participants with a risk or established diagnosis of a chronic condition associated with low PA levels.^34,38,39^ Six studies reported on chronic health conditions,^29,30,35–37,39^ with 2 of these reporting on multimorbidity.^30,37^ Smoking status was recorded in 5 studies,^28,29,34,35,37^ with active smokers ranging from 5% to 18%.

Of the 101 practices recruited to the studies under review, only 1 study reported that they included a rural practice.^30^ Nine studies included data on ethnicity.^28,32–39^ However, 4 studies had over 90% White participant.^32–35^ More recent studies had higher proportions of ethnic minorities, with Harris et al.’s 2018 study reporting 23%,^37^ and Khunti et al. reporting 28%.^39^ The latter study incorporated a strategy of reduced lower age limit for participants from ethnic minority groups at higher risk of diabetes. One study used “first language” as a measure of ethnicity and reported 34 different first languages, but the vast majority (86%) of participants were White.^36^ Ten studies reported employment, but in different ways: 7 reported on retirement,^32–35,37–39^ with retired people constituting a range of 29%^37^ to 85%^32^ of the respective study populations. Two studies recorded income or type of employment: Petrella et al. reported that 19% of participants were on very low incomes,^30^ and of the participants in Harris et al.’s 2018 study, 14% had past or current manual jobs.^37^ Of the 8 studies that recorded relationship status,^29,30,32,33,35,37–39^ only 1 reported a minority of single participants.^32^ Data on whether participants were living alone or with others, e.g. carers or family members, was not reported in any paper. Level of education attained was collected in 7 studies.^28,30,32,33,35,38,39^ At least one-third of participants had left education at or prior to completion of secondary school in 4 studies,^28,30,32,33^ while in 3 studies, at least 40% of participants had a third-level qualification.^37–39^

### Recruitment strategies

The primary mode of participant recruitment, with 1 exception,^30^ was a 2-step process, involving: (i) chart or database search to identify people that met the inclusion criteria, followed by (ii) a letter sent out from the practice to the patient. The letter usually contained an outline of the study and details on how to register an interest; some letters also contained a screening form.^28,31^ One study employed a combination of letters and phone calls.^32^ Nonrespondents were followed-up in 2 studies, by letter after 6 weeks^35^ and by telephone from the GP after 12 weeks.^31^ Petrella et al. did not utilize letters and are the only study to have 2 distinct strategies within general practice: firstly through opportunistic invitation during the practice visit and secondly by telephone call from the GP to potential participants, identified from the practice database.^30^ Two separate recruitment strategies were also employed by Khunti et al., drawing on participants identified from research databases as well as from practice databases.^39^ Support for the practices from the research team was reported in 9 studies, and involved training of practice teams and research assistants helping with database searches.^29,31–37,39^ Seven practices reported on the duration of recruitment,^30,32,33,35–37,39^ which ranged from 2^37^ to 27^36^ months. Iliffe et al. took 9 months longer than anticipated because of the need to recruit more practices at both sites and to allow more time at each practice to undertake recruitment.^36^

### Recruitment efficiency

Three studies reported the size of the population that could be invited (pool), thereby facilitating a calculation for efficiency A.^31,35,37^ Efficiency B was calculated for each study as all 12 studies provided data on the number invited to participate. The 2 studies that invited the least numbers had the highest efficiency B.^30,31^ One study failed to provide data on the number attending screening,^35^ and efficiency C was calculated for the others. High efficiency C scores were noted among both large^39^ and small^30^ studies.

## Discussion

This systematic review examined the reporting of demographic and recruitment details from RCTs of PA in general practice involving adults aged 45 years and older. It included 12 studies, all of which were conducted in high-income countries, including 101 participating practices and a combined total of 6,042 participants, 57% of whom were female. The quality of participant demographic reporting was mixed, with 6 studies awarded a score of 7/10 or above. However, high reporting quality did not equate with diverse participation; e.g. Harris et al.’s 2018 study scored 9/10 but 64% of participants were female,^37^ and while Kolt et al.’s 2012 paper scored 8/10, 97% of participants were White New Zealanders.^33^ Lack of consistency regarding how employment, income, and education level are reported limits analysis on socioeconomic profile of the trials.

Regarding the first specific objective of this review, it is evident that while participant demographics are being reported in trials, the reporting style varies between studies, and this hinders analysis of the data. Socioeconomic status is reported in several different ways: whether one is working or not does not differentiate between socioeconomic groups; few studies contained detail on income level or category of work. Similarly, retirement is reported in 7 studies, but this category does not indicate socioeconomic group. The high levels of retirement in some of the study populations reported may be a factor in successful recruitment, as lack of time has been identified as an important factor in nonparticipation in trials of PA in this setting.^40^ Furthermore, an important finding of this review has been the disconnect between reporting of demographics and demographic spread—some of the studies with the highest reporting scores had very low percentages of ethnic minorities.^32^ Research suggests that older adults who are already physically active and healthy are more likely to participate in a study designed to improve PA^41^ and that less healthy adults are reluctant to participate.^42^ However, this review reports that 6 of the studies only accepted participants that were already inactive, and across the 7 studies that reported on chronic illness, most participants had at least 1.

For the second objective, to examine recruitment, this review reports that approaches to recruitment across studies are similar. Recruitment to trials in general practice is based on database review to identify participants followed by letters sent from the practice to invite selected individuals; this was observed across the studies with 1 exception: Petrella et al. employed a combination of opportunistic face-to-face invites and telephone calls.^30^ Research indicates that combining recruitment strategies (active, such as directly inviting patients, and passive, including posters, letters, and media advertisements) may be optimal, providing a wider demographic base that is more representative of the inactive population at risk of health problems and a reasonable recruitment rate.^43,44^ For optimal recruitment, the GP and/or other members of the clinical practice team must be involved in “both the design and conduct” of the recruitment.^45^

Reporting of recruitment lacked detail—only 6 studies outlined how practices were recruited.^29,30,33,36,37,39^ This is consistent with other research which stated that it is not possible to determine which strategies are optimal for recruitment, especially for harder to reach groups, due to insufficient reporting.^46^ However, for the third objective of this review, most studies provided enough detail to calculate efficiency of recruitment but only 2 provided enough detail for calculation of all efficiency ratios.^31,37^ The studies with higher recruitment efficiency ratios each employed more than 1 recruitment strategy: Khunti et al. used research databases as well as practice databases, and allowed a lower age limit for ethnic groups at high risk of developing diabetes^39^; opportunistic face-to-face invitations and telephone calling were utilized by Petrella et al.^30^; and a sample of nonrespondents were telephoned after 3 weeks in the study by Tully et al.^31^

### Strengths and limitations

The review was restricted to RCTs conducted in general practice settings; studies conducted in other primary care or community settings were excluded. A rigorous and systematic approach was taken to quality assessment of recruitment, which was the focus of the research aim. The exclusion of studies that were published in languages other than English restricted the review to 5 countries. On the other hand, these countries share similar systems of general practice, thereby making the context more homogeneous. Furthermore, this systematic review was designed to examine the demographic characteristics reported for each study population; measuring and comparing to the general population in the trial settings was beyond the scope of the study and the authors, therefore, cannot make conclusions about sample representativeness.

### Implications for research and/or practice

The findings of this review suggest that future research should aim for consistency of reporting populations recruited to PA trials from general practice. Important participant data are currently not being reported. Based on this review, the authors recommend that journals encourage researchers to record and report data relating to the following: employment and educational status as well as income; ethnicity; morbidity, including specific diagnoses and number of regular medications; whether living alone; and whether participants are full-time carers. Second, future research in general practice settings should involve GPs as stakeholders as early as possible in the design process. Buy-in from GPs and their teams should ensure more effective recruitment and inclusion of harder to reach groups. Acknowledging the contribution of GPs to the research process and rewarding it appropriately has been reported as an important factor in research participation.^47^ Generalizability of trial results hinges on optimum recruitment that generates both adequate numbers and a representative sample size.

## Conclusion

It is imperative that researchers of PA interventions aimed at adults aged 45 years and above in general practice report demographic details and recruitment factors in a consistent way. This systematic review has presented deficits in the reporting of both demographics and recruitment. Future research should aim for a standardized approach to reporting.

## Supplementary Material

cmac128_suppl_Supplementary_Material

cmac128_suppl_Supplementary_Checklist

## Data Availability

The data underlying this article are available by request to the corresponding author.
