# Supplementary material for: Recruitment and characteristics of participants in trials of physical activity for adults aged 45 years and above in general practice: a systematic review
Source: Fam Pract. 2022 Dec 6;40(2):387–97. doi: 10.1093/fampra/cmac128 (PMC10047612; doi:10.1093/fampra/cmac128)
Supplement: cmac128_suppl_Supplementary_Material [file cmac128_suppl_supplementary_material.docx]

**Supplementary Data**

**Supplementary data 1: Search strategy**

1. **Population of Interest, Intervention, Comparator, Outcome**

| **Population of interest:** | Adults – (45 years and above) |
| --- | --- |
| **Intervention:** | Physical Activity delivered in General Practice settings |
| **Comparator:** | Control group with no intervention, alternative Physical Activity interventions |
| **Outcome:** | Increased Physical Activity levels or improved health; focusing on the recruitment strategies described |

1. **Search**

**Terms:**

- family practice/ general practice/ primary care AND physical activity/exercise AND adult/older adult AND randomized controlled trial/ RCT
- (family practice OR general practice OR primary care) AND (physical activity OR exercise) AND (adult OR older adult)
- (family practice OR general practice OR primary care) AND (physical activity OR exercise) AND (adult OR older adult) AND (recruit* strateg*)
- (family practice OR general practice OR primary care) AND (physical activity OR exercise) AND (adult OR older adult) AND (recruit*)

**Supplementary data 2: Description of studies and interventions**

| **Author,**  **year,**  **location** | **Study aim,**  **study design** | **Intervention** | **Population,**  **setting** | **Outcome** | **Duration, follow-up** |
| --- | --- | --- | --- | --- | --- |
| **Stevens et al**  1998,  United Kingdom | To increase PA of inactive people in primary care.  2-arm RCT | Assessment with exercise professional and a 10-week personalised programme | Patients aged 45–74 years from two general practices in West London (N=714) | At 8 months there was an increase in activity level in the intervention group and similar but smaller changes in the control group. | Duration 10 weeks,  follow-up 8 months |
| **Halbert et al**  2000,  Australia | To increase self-reported PA, walking time and vigorous exercise.  2-arm RCT | Referral for exercise advice from an exercise specialist | Patients aged ≥60 from two general practices in Adelaide, Australia (N=299) | PA increased significantly from baseline levels in both intervention and control groups. Men in the intervention group had the greatest levels of improvement in frequency of vigorous exercise compared to control. Effects were maintained at 6 and 12 months. | Duration 3 months,  follow-up 6 and 12 months |
| **Petrell et al**  2003,  Canada | To increase self-reported aerobic fitness and exercise self-efficacy.  2-arm RCT | Exercise prescription | Patients aged ≥65 from four general practices in Ontario (N=241) | An increase in aerobic capacity was observed at 6 and 12 months. | Duration and follow-up 3, 6 and 12 months |
| **Tully et al**  2005,  Northern Ireland | To increase self-reported walking time.  2-arm RCT | Advice delivered by GP to walk briskly for 30 minutes five days per week for 12 weeks | Low active adults aged 50–65 years from three urban general practices in Northern Ireland (N=31) | At 12 weeks, the mean distance walked was significantly higher in the walking group compared to the control group. | 3 months,  no follow-up |
| **Kolt et al,**  2007,  New Zealand | Increase minutes of self-reported PA.  2-arm RCT | Eight telephone counselling sessions delivered by a trained exercise counsellor | Low active adults aged ≥65 from three general practices in Auckland (N=186) | Moderate leisure PA increased by 86.8 minutes per week more in the intervention group.  More participants in the intervention group reached 2.5 hours of MVPA per week after 12 months. | Duration 3 months,  follow-up 6 and 12 months |
| **Kolt et al,**  2012,  New Zealand | To increase weekly self-reported leisure time.  2-arm RCT | Green prescription with pedometer | Low active adults aged ≥65 from 10 general practices in Auckland  (N=330) | For both groups, there were significant increases across all PA domains at 3 months (end of intervention) that were largely maintained after 12 months of follow-up. | Duration 3 months,  follow-up 12 months |
| **Devi et al,**  2014,  United Kingdom | To increase device-measured step count.  2-arm RCT | Cardiac rehabilitation programme delivered by study team researcher | People with angina from nine general practices in the Coventry and Warwickshire region of England (N=94) | At 6 weeks, the intervention group had greater improvements in step count. There was no significant improvement in daily steps at 6 months in the intervention group. | Duration 6 weeks,  follow-up 6 months |
| **Harris,**  2015,  United Kingdom | Increase device-measured time spent in MVPA and step count.  2-arm RCT | Walking intervention, delivered by practice nurse | People aged 60–75 years from three general practices in Oxfordshire and Berkshire (N=298) | At 3 months changes in both average daily step-counts and weekly MVPA in ≥10-minute bouts were significantly higher in the intervention than control group  At 12 month follow-up the differences were maintained. | Duration 3 months,  follow-up 12 months |
| **Iliffe et al,**  2015,  United Kingdom | Increase self-reported MVPA  3-arm RCT | Home and class-based exercise programmes delivered by instructors and peer mentors. | People aged 65 years and over from 43 general practices in London, Nottingham and Derby (N=1,256) | 49% of class-based group reached the MVPA target compared with 38% from usual care.  Differences between class-based and usual care persisted 24 months after intervention. | Duration 12 months,  follow-up 18 and 24 months |
| **Harris et al,**  2018,  United Kingdom | To increase steps per week and minutes of device-measured MVPA.  3-arm RCT | 12-week walking programmes, diaries, nurse support | People aged 45–75 years, from seven general practices in London, UK (N=1,023) | PA increased significantly at 12 months in both intervention groups compared with control group, with no difference between interventions.  Intervention effects persisted at 3-year follow-up. | Duration 12 months,  follow-up 36 months |
| **Peacock et al,** 2020  United Kingdom | To increase device-measured PA levels.  2-arm RCT | Personal PA feedback through a digital system and web-app for 3 months and five health trainer-led sessions | People at medium or high risk for cardiovascular disease and/or type II diabetes aged 40–70 years from six general medical practices in southwest of the UK (N=204) | At 3-month and 12-month follow-up the mean PA levels, in multiple dimensions of PA, were statically equivalent for the intervention and control groups | Duration 3 months, follow-up 12 months |
| **Khunti et al,** 2021, United Kingdom | To increase device-measured step count and time spent in MVPA.  3-arm RCT | A group-based behavioural PA intervention, delivered alone or with supporting text messages and telephone calls. | Patients aged 40–74 years for Caucasian, or 25–74 years for ethnic minority patients to account for higher diabetes risk. (N=1366) | At 12 months, intervention group had an extra step count of 547 steps/day compared to the control, and were 1.61 times more likely to achieve 150 min/week of MVPA. At 48 months, differences were not sustained | Duration 12 months, follow-up 24 and 48 months |

**Abbreviations: PA, physical activity; MVPA, moderate-to-vigorous physical activity; RCT, randomised control trial.**
